# A novel biphenyl urea derivate inhibits the invasion of breast cancer through the modulation of CXCR4

**DOI:** 10.1111/jcmm.12536

**Published:** 2015-03-08

**Authors:** Yingzhuan Zhan, Han Zhang, Jing Li, Yanmin Zhang, Jie Zhang, Langchong He

**Affiliations:** a School of Pharmacy, Health Science Center, Xi’an Jiaotong UniversityXi’an, Shaanxi Province, China

**Keywords:** breast cancer, biphenyl urea derivate, invasion, CXCR4

## Abstract

The increased migration and invasion of breast carcinoma cells are key events in the development of metastasis to the lymph nodes and distant organs. CXCR4, the receptor for stromal-derived factor-1, is reportedly involved in breast carcinogenesis and invasion. In this study, we investigated a novel biphenyl urea derivate, TPD7 for its ability to affect CXCR4 expression as well as function in breast cancer cells. We demonstrated that TPD7 inhibited the breast cancer proliferation and down-regulated the CXCR4 expression on breast cancer cells both over-expressing and low-expressing HER2, an oncogene known to induce the chemokine receptor. Treatments with pharmacological proteasome inhibitors partial suppressed TPD7-induced decrease in CXCR4 expression. Real-time PCR analysis revealed that down-regulation of CXCR4 by TPD7 also occurred at the translational level. Inhibition of CXCR4 expression by TPD7 further correlated with the suppression of SDF-1α-induced migration and invasion in breast tumour cells, knockdown of CXCR4 attenuated TPD7-inhibitory effects. In addition, TPD7 treatment significantly suppressed matrix metalloproteinase (MMP)-2 and MMP-9 expression, the downstream targets of CXCR4, perhaps *via* inactivation of the ERK signaling pathway. Overall, our results showed that TPD7 exerted its anti-invasive effect through the down-regulation of CXCR4 expression and thus had the potential for the treatment of breast cancer.

## Introduction

Chemokines are expressed by many tumour types and can promote mitosis, modulate apoptosis, survival, and angiogenesis 1,2. Stromal cell-derived factor-1(SDF-1 or CXCL12), which belongs to the CXC chemokine subfamily, is produced in two forms, SDF-1α (CXCL12α) and SDF-1β (CXCL12β), by alternative splicing of the SDF-1 gene. Interaction between the chemokine receptor CXCR4 and its ligand, SDF-1α, have also been shown to be involved in metastasis of several tumours 3–7. CXCR4 over-expressing in human breast cancer tissues was linked to the nodal spread of breast cancer. In addition, the receptor has also been associated with metastatic disease and poor disease free survival 3. The SDF-1α-CXCR4 interaction promotes tumour progression by several possible mechanisms. CXCR4/SDF-1 interactions trigger the activation of many downstream pathways, including Ca^2+^ influx, activation of the MAPK/ERK-1/2 pathway, activation of phosphatidylinositol 3-kinase and Akt, as well as increased NF-κB (nuclear factor kappa-light-chain-enhancer of activated B cells) activity 8–11. Because of its involvement in both metastasis and primary tumour growth, CXCR4 is an ideal target to investigate novel therapeutic interventions. Some studies have successfully shown that blockade of CXCR4 or CXCR4/SDF-1 interactions by siRNA and chemical inhibitors suppressed cancer cell proliferation, invasion and metastasis.

**Figure 1:**
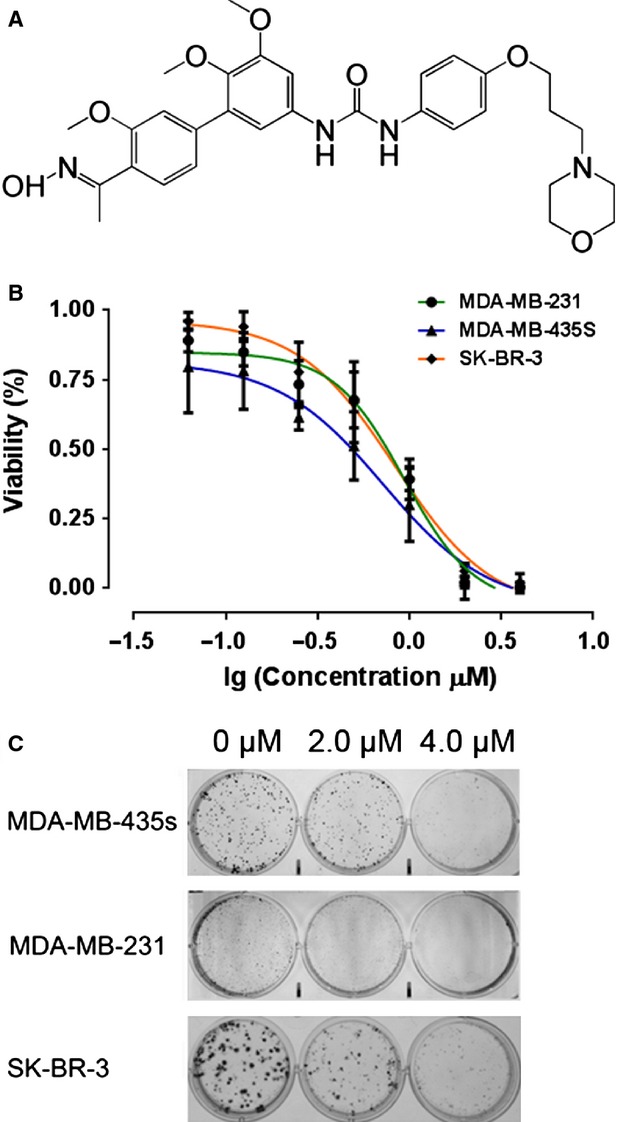
TPD7 suppressed breast cancer cell proliferation and colony formation. (A) Chemical structure of TPD7. (B) Cells were treated by TPD7 at indicated concentrations for 48 hrs. TPD7 inhibited MDA-MB-435s, MDA-MB-231 and SK-BR-3 cells growth in a dose-dependent manner. (C) Effect on colony formation of MDA-MB-435s, MDA-MB-231 and SK-BR-3 cells by TPD7. TPD7 showed significant inhibition on the colony formation of all cell lines we tested. Data were represented as the means ± SEM from three repeated experiments.

In the present studies, we investigated the effect of TPD7 (*N*-(4′-acetyl-3′,5,6-trimethoxybiphenyl-3-yl)-*N*′-[4-(3-morpholin-4-ylpropoxy)phenyl]urea) as a novel regulator of CXCR4 expression and function in breast cancer. TPD7 was a novel biphenyl urea taspine derivatives designed and synthesized using dissection strategies in our laboratory 12 (Fig.1A). Taspine was a natural alkaloid originally identified by screens of *Radix et Rhizomaleonticis* (Hong Mao Qi in Chinese) using cell membrane chromatography 13. It has many pharmacologic actions such as bacteriostasis, antibiosis, antivirus, anti-inflammatory, antiulcer effects 14–16. Previously, we found that taspine displayed anticancer and antiangiogenesis properties 17,18. As one of taspine derivatives, TPD7 displayed significant inhibitory activity on proliferation of several different cancer cell lines. Because CXCR4 was known to mediate proliferation, invasion and metastasis of tumour cells, in this study, we investigated whether TPD7 could modulate the expression of CXCR4 and thus inhibited breast tumour cell proliferation and invasion.

## Materials and methods

### Reagents

TPD7 was from the Research and Engineering Center for Natural Medicine, Xi’an Jiaotong University. LEIBOVITZ’S L15 medium, MG132 and chloroquine were purchased from Sigma-Aldrich (St. Louis, MO, USA). Foetal bovine serum (FBS) was obtained from Lanzhou national hyclone Bio-Engineering Co., Ltd (Lanzhou, China). Recombinant human SDF-1α was purchased from PeproTech (Rocky Hill, NJ, USA). Antibodies against CXCR4 were obtained from Abcam (Burlingame, CA, USA). Matrix metalloproteinase (MMP)-2 rabbit mAb and MMP-9 rabbit mAb were obtained from Epitomics (Burlingame, CA, USA). p44/42 MAPK (ERK1/2) rabbit mAb and p-p44/42 MAPK (p-ERK1/2) rabbit mAb were purchased from Cell Signaling (Danvers, MA, USA). Horseradish Peroxidase (HRP)-conjugated Glyceraldehyde-3-phosphate dehydrogenase (GAPDH) monoclonal antibody was from Proteintech Group (Chicago, IL, USA). Total RNA extraction kit was from Fastagen (Fastagen, Shanghai, China). PrimeScript RT Master Mix Perfect Real Time Kit (DRR036A) and SYBR Premix Ex Taq II were from TaKaRa (Dalian, China). Lipofectamine 2000 was from Invitrogen (Carlsbad, CA, USA). Other reagents used were analytical grades.

### Cell culture

MDA-MB-435s, MDA-MB-231 and SK-BR-3 breast cancer cell lines were obtained from Shanghai Institute of Cell Biology in the Chinese Academy of Sciences in 2012. MDA-MB-435s and MDA-MB-231 cells were maintained in LEIBOVITZ’S L15 medium supplemented with 10% (v/v) FBS, SK-BR-3 cells were cultured in DMEM supplemented with 20% FBS. All cell lines were incubated at 37°C in a 5% CO_2_ incubator with saturated humidity.

### Cell proliferation assay

MDA-MB-435s, MDA-MB-231 and SK-BR-3 cells were cultured in 96-well plates, and fresh medium with TPD7 (0, 0.625, 1.25, 2.50, 5.00, 10.0, 20.0, 40.0 μM), was added for 48 hrs. Cell proliferation reagent MTT was added and incubated at 37°C and 5% CO_2_ for 4 hrs. Absorbance was then measured at 490 nm with a microplate reader (Bio-Rad Instruments, Hercules, CA, USA).

### Colony formation assay

MDA-MB-435s, MDA-MB-231 and SK-BR-3 cells were cultured in 6-well plates and fresh medium with or without TPD7 were added for 10–15 days. Colonies with cell numbers of >50 cells per colony were counted after staining with crystal violet solution. All the experiments were performed in triplicate wells in three independent experiments.

### Western blot analysis

The MDA-MB-435s and MDA-MB-231 cells treated with or without TPD7 for 48 hrs were prepared by extracting proteins with RIPA lysis buffer containing protease inhibitor cocktail and phosphatase inhibitor cocktail on ice. Cell lysates were analysed for Western blot analysis with primary antibodies, followed by enhanced chemiluminescence. Blots were reprobed with GAPDH to compare protein load in each lane.

### RNA extraction and PCR analysis

Total RNA of MDA-MB-435s and MDA-MB-231 cells treated with or without TPD7 were isolated using total RNA extracted kit according to the manufacturer’s protocol. The RT-PCR was performed with PrimeScript RT Master Mix Perfect Real Time kit (TaKaRa DRR036A). Real-time PCR was performed with SYBR® Premix Ex TaqTM II and a Thermal Cycle Dice Real time system (TaKaRa). The result was analysed using the manufacturer’s program (Thermal Cycler Dice™ Real Time System). The primer sequences were as following: GAPDH forward primer: 5′-GCACCGTCAAGGCTGAGAAC-3′; GAPDH reverse primer: 5′-TGGTGAAGACGCCAGTGGA-3′; CXCR4 forward primer: 5′-CCTGCCTGGTATTGTCATCCTG-3′; CXCR4 reverse primer: 5′-ACTGTGGTCTTGAGGGCCTTG-3′. Melt curve analysis was performed at the end of each PCR to confirm the specificity of the PCR product. Threshold cycle (Ct) values of CXCR4 in each sample were normalized with the GAPDH expression.

### Wound healing assay

MDA-MB-435s and MDA-MB-231 cells were planted into 6-well plate and allowed to grow to 70% confluency in complete medium. Cells were then serum starved for 24 hrs, and cell monolayers were scratched with a pipette tip. Wounded monolayers were then washed several times with serum-free medium to remove floating cells and photographed in microscope. Cells were incubated in medium in the absence or presence of TPD7 for 48 hrs. After incubation, the growth medium was then changed to basal medium with or without 100 ng/ml SDF-1α. After 24 hrs, cell migrating into the wound surface and the average distance of migrating cells was determined under an inverted microscope.

### Invasion assay

Cancer cells were suspended in medium and seeded into the Millicell chambers with polycarbonate membranes of 8-μm pore size coated with 100 μl 1 mg/ml Matrigel (Becton Dickinson, Franklin Lakes, NJ, USA). After preincubation with or without TPD7 for 48 hrs, Millicell chambers were then placed into 24-well plates in which were added the basal medium only or basal medium containing 100 ng/ml SDF-1α. After incubation, the upper surface of Millicell chambers was wiped off with a cotton swab and invading cells were fixed with 100% methanol and then stained with 0.2% crystal violet (Beijing Chemical Works, Beijing, China). The invading cell numbers were counted in five randomly selected microscope fields (×100).

### siRNA analysis

A double-stranded siRNA against CXCR4 and nonspecific siRNA (control siRNA) were obtained from Shanghai GenePharma Co., Ltd. (Shanghai, China) MDA-MB-435s and MDA-MB-231 cells were seeded in a 6-well plate and transfected with the siRNA against CXCR4 for 24 hrs at a final concentration of 50 nM with Lipofectamine 2000 reagent (Invitrogen) according to the manufacturer’s instructions. Transfection with a control siRNA was served as a negative control. Cells were subjected to RT-PCR to detect gene expression and western blotting to protein expression. The transfected cells were seeded for invasion assays.

### Statistical analysis

All values are expressed as means ± SEM. Statistics was determined with anova. Results were considered statistically significant if the *P* < 0.05.

## Results

### TPD7 suppressed breast cancer cell proliferation and colony formation

To evaluate the effect of TPD7 on breast cancer cells, we observed its action on cell proliferation and colony formation. The results showed that TPD7 significantly inhibited cell proliferation of MDA-MB-435s, MDA-MB-231 and SK-BR-3 cells. The IC50 was 7.22 μM, 9.37 μM and 8.36 μM respectively (Fig.1A). In colony formation assay, upon 10–15 days continuous culture, TPD7 also suppressed colony formation of MDA-MB-435s, MDA-MB-231 and SK-BR-3 cells (Fig.1B). These findings indicated that TPD7 had potential anti-tumour properties in different type breast cancer cell lines.

### TPD7 suppressed the expression of CXCR4 protein in breast cancer cells

**Figure 2:**
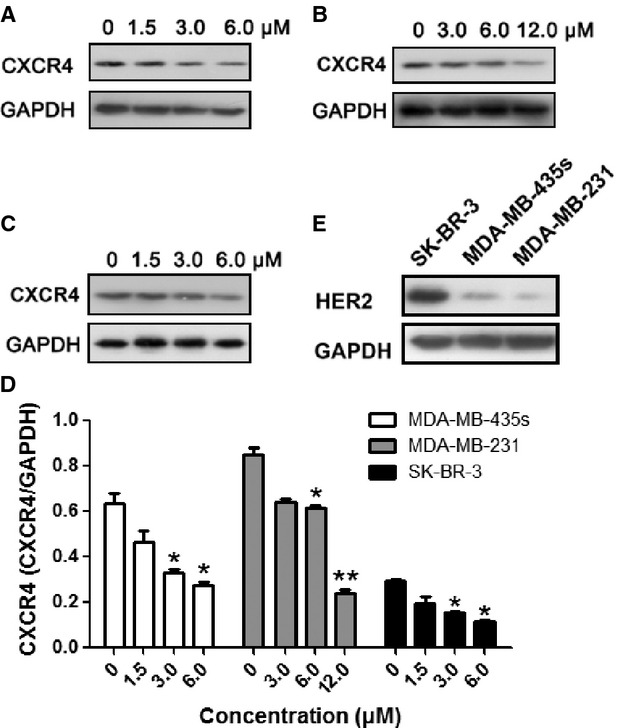
TPD7 down-regulated CXCR4 in different breast cancer cell lines. MDA-MB-435s (A), MDA-MB-231 (B) and SK-BR-3 (C) cells were incubated with TPD7 at different concentrations for 48 hrs. Whole-cell extracts were prepared and analysed by Western blot analysis with antibodies against CXCR4. The same blots were stripped and reprobed with GAPDH antibody to show equal protein loading. (D) Quantification of A, B and C. (E) The expression level of HER2 in SK-BR-3, MDA-MB-435s and MDA-MB-231 cells. The results shown were representative of three independent experiments. *P < 0.05; **P < 0.01 compared with untreated control cells.

We investigated the expression level of CXCR4 in the three breast cancer cell lines, namely SK-BR-3, MDA-MB-435s and MDA-MB-231, and found that CXCR4 protein expression was down-regulated by the TPD7 in a dose-dependent manner at the concentrations used in the experiments (Fig.2A–D). HER2 has been shown to induce the expression of CXCR4 in breast cancer cells 19, and it has been reported to regulate the expression of CXCR4 by stimulating CXCR4 translation and attenuating CXCR4 degradation 20. We also examined whether TPD7 affected CXCR4 expression related to status of HER2 expression in breast cancer cells. We first investigated the expression level of HER2 in the above three different breast cancer cell lines. As shown in Figure2, the results indicated that SK-BR-3 had high levels of HER2 expression as shown in western blotting, whereas MDA-MB-435s and MDA-MB-231cells had lack of HER2 expression (Fig.2E), which were consistent with the previous reports 21–24. In this experiment, down-regulation of CXCR4 expression were observed in all above cell lines, suggesting that the reduced expression of CXCR4 by TPD7 was irrespective of HER2 status in breast cancer.

### Down-regulation of CXCR4 by TPD7 was partial mediated through its degradation

**Figure 3:**
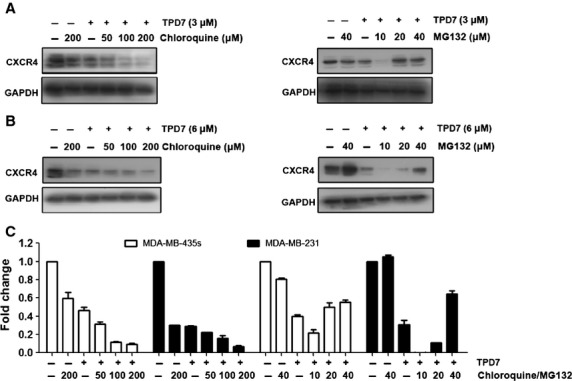
TPD7 suppressed CXCR4 by proteasomal degradation. MDA-MB-435s (A) and MDA-MB-231 (B) cells were treated with indicated concentrations of chloroquine or MG132 for 1 hr, followed by treatment of TPD7 for 48 hrs. Whole-cell extracts were prepared and analysed by western blot analysis using antibodies against CXCR4. The same blots were stripped and reprobed with GAPDH antibody to show equal protein loading. (C) Quantification of A and B. The results shown were representative of three independent experiments.

Because TPD7 could down-regulate CXCR4 expression, and CXCR4 has been shown to undergo ligand-dependent lysosomal degradation 25. We next examined the ability of chloroquine, a lysosomal inhibitor, to block TPD7-induced degradation of CXCR4. The cells were pre-treated with chloroquine for 1 hr before exposure to TPD7 for 48 hrs. Our results showed that chloroquine had no effect on TPD7-induced degradation of CXCR4 (Fig.3), suggesting that this was an unlikely basis for the suppression of TPD7 on CXCR4 expression.

Since CXCR4 has also been shown to undergo ubiquitination at its lysine residue followed by degradation 25,26, we next investigated whether TPD7 induced down-regulation of CXCR4 through proteasomal degradation. To determine this, we examined the ability of MG132, a proteasome inhibitor, to block TPD7-induced degradation of CXCR4. MDA-MB-435s and MDA-MB-231 cells were pre-treated with MG132 for 1 hr before being exposed to TPD7 for 48 hrs. As shown in Figure3, MG132 could prevent TPD7-induced degradation of CXCR4, suggesting that this is one pathway for suppression of expression of CXCR4.

### TPD7 down-regulated CXCR4 mRNA expression

**Figure 4:**
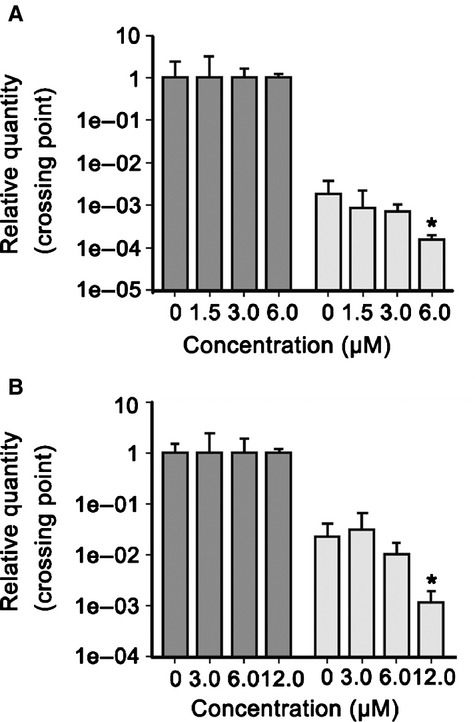
TPD7 suppressed CXCR4 mRNA level in breast tumour cells. MDA-MB-435s (A) and MDA-MB-231 (B) cells were treated with TPD7 for the indicated concentrations for 48 hrs, and then real-time PCR was performed to measure the relative quantities of CXCR4 mRNA, with GAPDH as endogenous control for measurement of equal loading of RNA samples. The results shown were representative of three independent experiments. *P < 0.05 compared with untreated control cells.

We further investigated whether suppression occurred at the transcriptional level using RT-PCR and also by quantitative PCR (real-time PCR). Cells were treated with TPD7 for different concentrations and then examined for steady-state mRNA level of CXCR4. As shown in Figure4, TPD7 induced down-regulation of CXCR4 mRNA expression in both MDA-MB-435s and MDA-MB-231 cells.

### TPD7 inhibited SDF-1α-induced breast cancer cell migration and invasion

**Figure 5:**
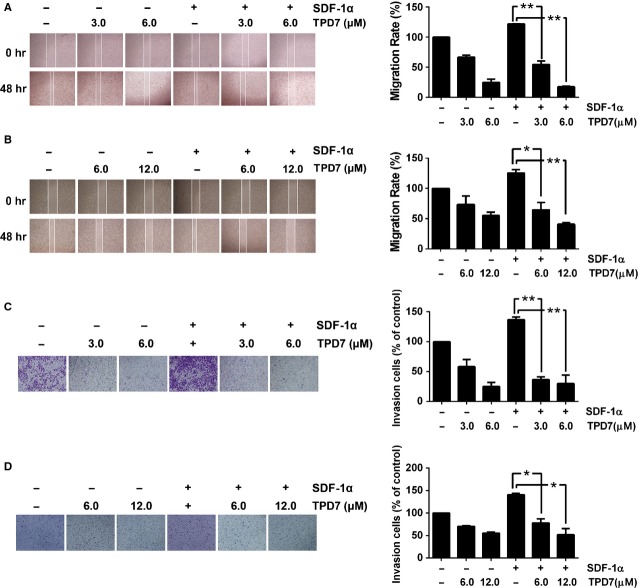
TPD7 suppressed migration and invasion of breast tumour cells. (A and B) Photographs and quantification of wound of cells treated with TPD7. Wound healing assay was performed for evaluating the inhibitory effect of TPD7 on MDA-MB-435s (A) and MDA-MB-231 (B) cell migration. Confluent monolayers of cells were scarred, and pre-treatment with TPD7 for 48 hrs before being exposed to 100 ng/ml SDF-1α for 24 hrs. The average distance of migrating cells into the wound surface were determined under an inverted microscope. The representative photographs showed the same area at time zero and after 48 hrs of incubation. (C and D) Photographs and quantification of the cell invasion through the Matrigel-coated polycarbonate membrane stained by 0.2% crystal violet. MDA-MB-435s (C) and MDA-MB-231 (D) cells were seeded in the top-chamber of the Matrigel. After pre-treatment with or without TPD7 for 48 hrs, Millicell chambers were then incubated with either the basal medium only or basal medium containing 100 ng/ml SDF-1α for 24 hrs. After incubation, they were assessed for cell invasion as described in Materials and methods. The representative photographs showed the cell migration through the polycarbonate membrane stained by 0.2% crystal violet. The results shown were representative of three independent experiments. *P < 0.05; **P < 0.01 compared with SDF-1α treated control cells.

The expression of CXCR4/SDF-1 in breast tumours has been correlated with a poor prognosis, increased metastasis 4. We found that both MDA-MB-435s and MDA-MB-231 cells migrated faster under the influence of SDF-1α and this effect was abolished on treatment with TPD7 (Fig.5A and B). To elucidate further the effect of TPD7 on SDF-1α induced cell invasion, we also found using an *in vitro* invasion assay, that treatment of TPD7 suppressed SDF-1α induced invasion of both MDA-MB-435s and MDA-MB-231 cells (Fig.5C and D).

### CXCR4 was a target of the inhibitory effect of TPD7 on cell invasion

**Figure 6:**
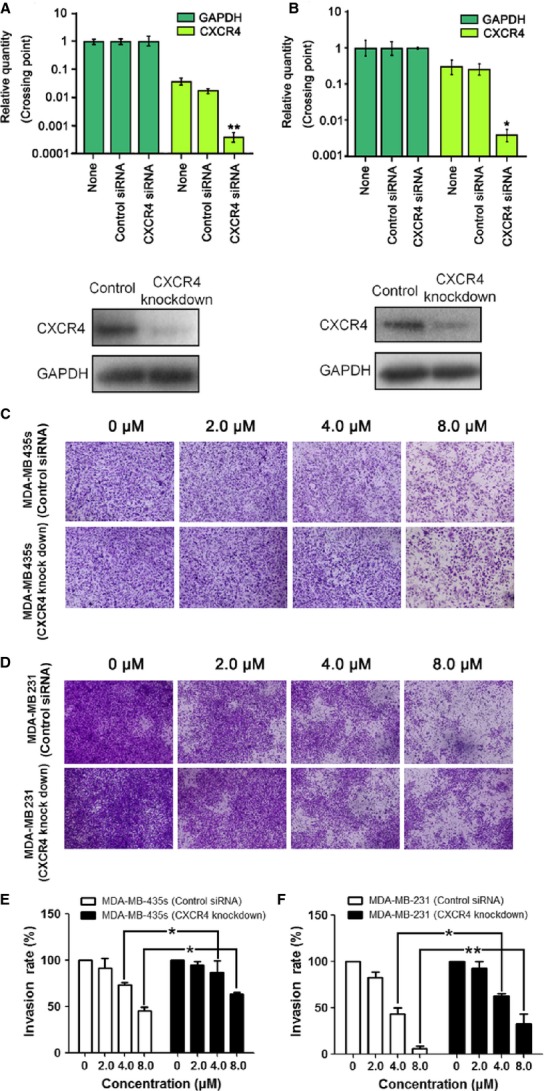
Effect of TPD7 on the invasion of cells transfected with siRNA of CXCR4 and wild-type cell *in vitro*. (A) RT-PCR showing mRNA (top) and Western blot analysis of CXCR4 protein expression (bottom) in MDA-MB-435s cells knockdown of CXCR4. (B) RT-PCR showing mRNA (top) and Western blot analysis of CXCR4 protein expression (bottom) in MDA-MB-231 cells knockdown of CXCR4. (C and D) MDA-MB-435s cells (C) and MDA-MB-231 cells (D) were transfected with siRNA against CXCR4 followed by treatment of TPD7 for 48 hrs at indicated concentration. Cell invasion was assessed by Millicell assay. (E and F) Quantification of C and D respectively. The results shown were representative of two independent experiments. Data were expressed as mean ± SEM. *P < 0.05, **P < 0.01 compared with cells transfected with control siRNA.

To further elucidate the effect of CXCR4 on TPD7’s cell invasion inhibition, we evaluated the effect on cell migration and invasion between wild tumour cells and CXCR4 knockdown cells, we observed the inhibition on cell mobility by wound healing assay and cell invasion by Millicell system. The results showed that treatment of TPD7 significantly decreased the migration and invasion ability of MDA-MB-435s and MDA-MB-231cells at the concentrations of 2.0–8.0 μM, respectively, and knockdown of CXCR4 by siRNA in the two cell lines significantly attenuated the inhibitory effects of TPD7 on migration and invasion, as compared with the control groups (Fig.6). It indicated CXCR4 was a key factor in the cell migration by TPD7.

### Effect of TPD7 on expression of MMP-2 and MMP-9 in breast tumour cells

**Figure 7:**
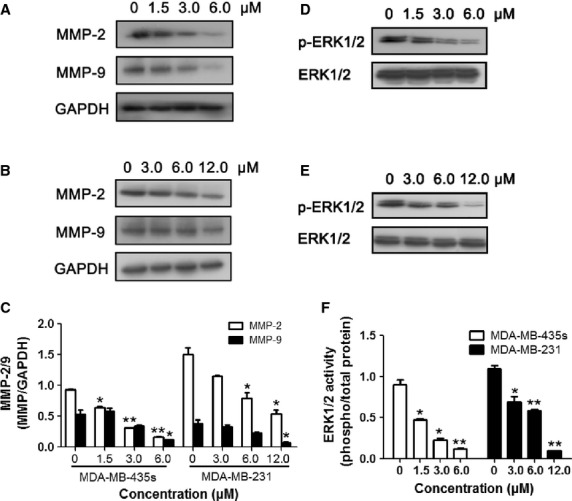
Effect of TPD7 on expression of MMP-2 and MMP-9 in breast tumour cells. (A and B) Western blot analysis of MMP-2 and MMP-9 protein expression in MDA-MB-435s cells (A) and MDA-MB-231 cells (B) after treatment with TPD7 at indicated concentrations for 48 hrs. (C) Quantification of A and B. (D and E) Western blot analysis of p-ERK1/2 protein expression in MDA-MB-435s cells (D) and MDA-MB-231 cells (E) after treatment with TPD7 at indicated concentrations for 48 hrs. (F) Quantification of D and E. The results shown were representative of three independent experiments. Data were expressed as mean ± SEM. *P < 0.05, **P < 0.01 compared with untreated control cells.

Previous studies has shown that binding of SDF-1α to CXCR4 plays a role in tumour metastasis by increasing invasion associated with MMP-9 and MMP-2 activation 20,27,28. MMPs are downstream targets of CXCR4-mediated signalling, CXCR4 promotes tumour migration and invasion through inducing expression of MMPs *via* the ERK signaling pathway 8,20,29. Therefore, we detected the effect of TPD7 on expression of phosphorylated ERK1/2, MMP-2 and MMP-9. As shown in Figure7, 48 hrs incubation with TPD7 reduced phosphorylated ERK1/2, MMP-2 and MMP-9 protein expression in a dose-dependent manner in MDA-MB-435s and MDA-MB-231 cells, suggesting that TPD7 perhaps suppressed the downstream targets of CXCR4, MMP-2 and MMP-9 *via* ERK pathway.

## Discussion

Metastases, rather than primary tumours, are responsible for breast cancer deaths, many experimental and clinical studies have demonstrated that SDF-1α/CXCR4 axis play a key role in regulating the directional migration of breast cancer cells to sites of metastasis. CXCR4 may be a useful prognostic indicator and a potential therapeutic target in cancer therapies in patients with breast cancer. In this study, we sought to elucidate whether the small molecule compound TPD7 could inhibit breast cancer growth and invasion, and demonstrated the biologically relevant modulation of oncogenic signaling by TPD7.

In this study, we firstly showed that TPD7 had significant inhibitory effect on breast cancer cell proliferation and invasion *in vitro*. We have been suggested that TPD7 treatment reduced CXCR4 signaling, and therefore cell migration and invasion. The expression of CXCR4 was investigated in breast cancer cells treated with TPD7. The epidermal growth factor receptor, c-erbB2, and its encoding gene, HER2/neu, have also been implicated in the positive regulation of CXCR4 expression at the post-transcriptional level 19,30. Our results indicated that TPD7 down-regulated the expression of CXCR4 not only in high HER2 expressing SK-BR-3 but also low HER2 expressing MDA-MB-435s and MDA-MB-231 cells. Because the reduced expression of CXCR4 by TPD7 was irrespective of HER2 status in breast cancer, we used two of these cell lines, MDA-MB-435s and MDA-MB-231, to gain insights into the mechanisms of TPD7 on down-regulation of CXCR4 in further studies.

Various reports suggest that expression of CXCR4 may be increased by inflammatory cytokines such as TNF 31 and VEGF 32. VEGF, a major angiogenic factor, is also a requisite autocrine factor for breast carcinoma invasion *in vitro*. Previous findings indicate that a VEGF autocrine pathway induces CXCR4 expression in breast carcinoma cells, thus promoting their directed migration towards specific chemokines 32. Here, we found that VEGF expression was minimally affected after TPD7 treatment (data not shown), thus suggesting that down-regulation of CXCR4 expression by TPD7 was not because of modulation of autocrine VEGF. Recently, more evidence has been presented to show the ligand-dependent down-regulation of the CXCR4 expression by lysosomal degradation 26, which involves atrophin-interacting protein 4-mediated ubiquitination and degradation 25. Our results, however, indicated that down-regulation of CXCR4 by TPD7 was induced not through lysosomal degradation but partial through proteasomal degradation, suggesting that proteasomal degradation was one pathway for suppression of CXCR4. In addition, we found that down-regulation of CXCR4 by TPD7 also occured at both the transcriptional and translational levels.

We also observed that TPD7 suppressed the ligand induced invasion of breast cancers, and this correlated with the down-regulation of CXCR4. To further validate this, knockdown of CXCR4 expression in MDA-MB-435s and MDA-MB-231 cells by siRNA significantly attenuated TPD7-inhibitory effects, thus suggesting that TPD7 had a potential to suppress tumour migration and invasion through its action on CXCR4.

The expression and activity of MMPs against matrix macromolecules have been linked to the development of malignant phenotypes 33 and the promotion of cell metastasis 34. MMP-2 and MMP-9, two major MMPs, play important roles in cancer cell invasion and metastasis 35,36. Recently, a growing body of evidence has been presented to show that SDF-1α/CXCR4 stimulation also leads to the secretion of various proteases, such as MMP-2 and MMP-9 by the breast cancer cells, which are then used to degrade the extracellular matrix, thereby facilitating cellular motility. These secreted proteases are also thought to facilitate the intravasation of cancer cells into the bloodstream. Furthermore, further studies show that MMPs are downstream targets of CXCR4-mediated signaling, CXCR4 regulation of MMPs expression is likely mediated by ERK signal pathways 28. In this study, we showed that MMP-2, MMP-9 and p-ERK1/2 activity was strongly decreased, which indicating that TPD7 inhibited MMP-2 and MMP-9 expression, the downstream targets of CXCR4, perhaps *via* inactivation of the ERK signaling pathway.

In summary, TPD7 inhibited breast cancer cell proliferation and invasiveness *in vitro*. The underlying mechanism was through down-regulating the expression of CXCR4, a key receptor involved in the cross-talk between tumour cells and its microenvironment, which contributed to its anti-invasive effects. Based upon these results, TPD7 might be promising candidate as CXCR4 inhibitor, the data supported further development of TPD7 as an adjuvant therapy agent for breast cancer. Further *in vivo* studies on the orthotopic breast cancer and metastasis to other organs are being planned to demonstrate the relevance of these observations to cancer treatment.
